# Association between fibrinogen and bone mineral density in postmenopausal women

**DOI:** 10.1186/s13018-023-03785-7

**Published:** 2023-05-22

**Authors:** Weibin Du, Zhenwei Wang, Yi Dong, Jintao Hu, Xiaoping Chen

**Affiliations:** 1 Research Institute of Orthopedics, The Affiliated Jiangnan Hospital of Zhejiang Chinese Medical University, Hangzhou, 312001 Zhejiang China; 2 Hangzhou Xiaoshan Hospital of Traditional Chinese Medicine, Hangzhou, 312001 Zhejiang China; 3 Shaoxing TCM Hospital Affiliated to Zhejiang Chinese Medical University, Shaoxing, 312000 Zhejiang China; 4 Orthopedics and Traumatology Department, Hangzhou TCM Hospital, Affilliated to Zhejiang Chinese Medical University, Hangzhou, 310000 Zhejiang China

**Keywords:** Fibrinogen, Bone mineral density, Postmenopausal women, Osteoporosis, NHANES

## Abstract

**Objective:**

There is very limited of evidence linking fibrinogen and bone mineral density (BMD) in postmenopausal women. Therefore, this study intended to examine the relationship between fibrinogen and total BMD in postmenopausal women.

**Methods:**

This cross-sectional analysis included 2043 postmenopausal women aged 50 years and older from the 1999 to 2002 National Health and Nutrition Examination Survey. The independent variable was fibrinogen and the outcome variable was total BMD. The association between fibrinogen and total BMD in postmenopausal women was examined using multivariate linear regression models, with subgroup analyses stratified by race. Smoothing curve fitting and generalized additive models further analyzed the sample data.

**Results:**

In multiple regression models adjusted for potential confounders, fibrinogen was negatively associated with total BMD (model 1: − 0.0002 [− 0.0002, − 0.0001], model 2: − 0.0000 [− 0.0001, − 0.0000], model 3: − 0.0001 [− 0.0001, − 0.0001]). In subgroup analysis stratified by race, fibrinogen levels were negatively associated with total BMD in postmenopausal women, Non-Hispanic Whites, and Mexican Americans. However, in Non-Hispanic Blacks, the correlation between fibrinogen levels and total BMD was not significant. For individuals that identify as Other Races, fibrinogen levels were positively correlated with total BMD.

**Conclusion:**

Our findings show a negative association between fibrinogen levels and total BMD in most postmenopausal women aged 50 years and older, however, is variable by race. In postmenopausal women, Non-Hispanic Whites and Mexican Americans, relatively high fibrinogen levels may be adverse to bone health.

## Introduction

Osteoporosis is a chronic disease involving a decrease in bone mineral density (BMD) and is one of the most prevalent factors contributing to fractures in the elderly [1]. The likelihood of osteoporosis increases with age [2], and the risk of osteoporosis increases further for women after menopause with changes in body hormone levels [3]. Postmenopausal osteoporosis has been acknowledged as a health care challenge, placing a burden on individuals and health care expenditures, and even posing a higher risk of death [4]. Therefore, postmenopausal osteoporosis in women should receive adequate attention.

More in-depth exploration of the risk factors associated with postmenopausal osteoporosis can help us in the early diagnosis, prevention and treatment of postmenopausal osteoporosis. Therefore, increasing number of biomarkers for bone health are being discovered, such as fibrinogen. Fibrinogen plays a key role in inflammation and coagulation as a core molecule of the coagulation response with inflammatory markers [5]. Inflammatory markers mechanistically promote bone resorption and inhibit bone formation, an imbalance that will lead to bone loss and increased fracture risk [6]. Recent studies have shown that inflammation, coagulation abnormalities, and vascular dysfunction may all contribute to impaired bone development and growth, whereas fibrinolytic enzymes inhibit inflammation and protect blood vessels by reducing fibrinogen accumulation [7]. This indirectly suggests that fibrinogen plays an essential role in maintaining bone health. In a cross-sectional study of 339 postmenopausal women, serum fibrinogen levels were found to be negatively correlated with femoral neck BMD [6]. Thus, fibrinogen may be one of the potential biological markers of bone mineral density. However, to date, evidence linking fibrinogen to BMD in postmenopausal women remains limited. Here, we analyzed data from the 1999 to 2002 National Health and Nutrition Examination Survey (NHANES) to explore the relationship between fibrinogen and BMD in postmenopausal women aged 50 years and older in the United States.

## Materials and methods

### Study population

We utilized data from the National Health and Nutrition Examination Survey (NHANES) from 1999 to 2002. A total of 2043 participants, who were postmenopausal women aged fifty years and older with complete data on fibrinogen and total BMD, were included in our study. For detailed methodology of NHANES, please visit http:// www.cdc.gov/nchs/nhanes/. This study was approved by the ethical review committee of the National Center for Health Statistics and each participant signed a written consent form.

### Study variables

The main variables in this study were fibrinogen (independent variable) and total BMD (dependent variable). Fibrinogen concentration in plasma was quantified by the Krauss coagulation method on the STA-Compact according to the National Institute of Standards and Technology (NIST) reference method. The test method involves measuring the rate of conversion of fibrinogen to fibrin in diluted samples under the influence of excess thrombin. Total BMD was measured by dual-energy X-ray bone densitometry. In addition, the following covariates are included: age, race, dietary intake within 24 h (protein, fat and dietary fiber), body mass index, poverty to income ratio, total cholesterol, total protein, blood urea nitrogen, and serum calcium.

### Statistical analysis

All estimates were calculated using sample weights according to the NCHS edited analysis guidelines. Weighted multiple regression analyses were performed to assess the independent association between fibrinogen and total BMD. The fibrinogen interquartile thresholds were 338, 386, and 441 (mg/dL). Three multivariate linear regression models were constructed: model 1, unadjusted for covariates; model 2, adjusted for age and race; and in model 3, adjusted for all covariates in Table 1. Further subgroup analyses were conducted. Weighted generalized additive models and smoothed curve fitting were used to address the nonlinearity of fibrinogen and total BMD. All analyses were performed using R (http://www.R-project.org) and EmpowerStats (http://www.empowerstats.com). Values of p less than 0.05 were considered statistically significant.

**Table 1:** Characteristics of the participants

Fibrinogen (mg/dL)	Total	Q1	Q2	Q3	Q4	P value
Age (years)	63.9 ± 10.3	61.3 ± 9.6	64.0 ± 10.1	64.4 ± 10.6	66.9 ± 10.5	< 0.0001
Race (%)						< 0.0001
Non-Hispanic Whites	77.5	82.4	78.7	76.0	71.0	
Non-Hispanic Blacks	8.7	4.9	7.0	11.0	13.3	
Mexican Americans	3.6	3.0	3.0	4.9	3.7	
Other Races (Other Race—Including Multi-Racial and Other Hispanics)	10.2	9.7	11.2	8.1	12.0	
*Dietary intake within 24 h*						
Protein (gm)	62.8 ± 28.0	67.5 ± 30.9	60.4 ± 23.0	62.3 ± 28.4	59.8 ± 28.0	< 0.0001
Fat (gm)	61.8 ± 32.7	64.9 ± 36.3	61.0 ± 30.0	62.4 ± 31.5	57.6 ± 31.4	< 0.0001
Dietary fiber (gm)	14.5 ± 8.6	15.4 ± 9.2	14.9 ± 8.8	14.1 ± 8.7	12.9 ± 7.1	< 0.0001
BMI (kg/m^2^)	28.7 ± 6.5	26.9 ± 5.4	28.2 ± 5.7	29.4 ± 6.4	31.1 ± 7.7	< 0.0001
Income poverty ratio	2.9 ± 1.5	3.3 ± 1.5	2.9 ± 1.5	2.9 ± 1.5	2.5 ± 1.4	< 0.0001
Blood urea nitrogen (mg/dL)	15.5 ± 6.0	15.2 ± 5.0	14.5 ± 4.9	15.4 ± 4.9	17.3 ± 8.6	< 0.0001
Total protein (mg/dL)	7.3 ± 0.5	7.3 ± 0.5	7.3 ± 0.5	7.4 ± 0.4	7.5 ± 0.5	< 0.0001
Total cholesterol (mg/dL)	214.9 ± 37.4	211.0 ± 36.3	215.9 ± 36.3	218.6 ± 36.4	215.2 ± 40.7	< 0.0001
Serum calcium (mg/dL)	9.48 ± 0.42	9.45 ± 0.42	9.48 ± 0.42	9.49 ± 0.41	9.50 ± 0.43	< 0.0001
Total BMD (g/cm^2^)	1.00 ± 0.12	1.03 ± 0.12	0.99 ± 0.11	1.00 ± 0.11	0.99 ± 0.12	< 0.0001
Fibrinogen (mg/dL)	383.6 ± 77.4	300.8 ± 30.8	361.2 ± 13.4	411.6 ± 15.7	495.3 ± 49.8	< 0.0001

Mean ± SD for continuous variables: the P value was calculated by the weighted linear regression model. (%) for categorical variables: the P value was calculated by the weighted chi-square test. BMD, bone mineral density

## Results

The weighted sociodemographic and medical characteristics of the participants are described in Table 1. A total of 2043 postmenopausal women aged 50 years and older were included in this study, and of these participants, Non-Hispanic Whites accounted for 77.5%, Non-Hispanic Black for 8.7%, Mexican Americans for 3.6%, and Other Races for 10.2%. In all four quartiles of fibrinogen (Q1–Q4), there were significant differences based on age, race, dietary intake within 24 h (protein, fat and dietary fiber), body mass index, poverty to income ratio, total cholesterol, total protein, blood urea nitrogen, phosphorus, and serum calcium (Table 1). Fibrinogen (quartiles, Q1–Q4) was significantly different from the group that included age in weighted multiple regression (Table 2); higher fibrinogen levels in each model were associated with lower BMD (g/cm^2^) (total BMD: model 1: − 0.0002 [− 0.0002, − 0.0001], model 2: − 0.0000 [− 0.0001, − 0.0000], model 3: − 0.0001 [− 0.0001, − 0.0001]). In a subgroup analysis stratified by race (Table 2), fibrinogen levels were negatively associated with total BMD in postmenopausal women, Non-Hispanic Whites and Mexican Americans, after controlling for potential confounders. However, the correlation between fibrinogen levels and total BMD was not significant in Non-Hispanic Blacks, and a significant positive correlation was found between fibrinogen levels and total BMD in those identifying with Other Races.

**Table 2:** The association between fibrinogen (mg/dL) and total bone mineral density (g/cm^2^)

	Model 1β (95% CI)	Model 2β (95% CI)	Model 3β (95% CI)
Fibrinogen (mg/dL)	− 0.0002 (− 0.0002, − 0.0001)	− 0.0000 (− 0.0001, − 0.0000)	− 0.0001 (− 0.0001, − 0.0001)
*Fibrinogen categories*			
Q1	0	0	0
Q2	− 0.0249 (− 0.0311, − 0.0188)	− 0.0101 (− 0.0156, − 0.0047)	− 0.0232 (− 0.0289, − 0.0174)
Q3	− 0.0343 (− 0.0407, − 0.0279)	− 0.0173 (− 0.0230, − 0.0116)	− 0.0398 (− 0.0458, − 0.0338)
Q4	− 0.0410 (− 0.0475, − 0.0345)	− 0.0100 (− 0.0158, − 0.0042)	− 0.0430 (− 0.0493, − 0.0368)
P for trend	< 0.001	0.006	< 0.001
*Stratified by race*			
Non-Hispanic Whites	− 0.0003 (− 0.0003, − 0.0002)	− 0.0001 (− 0.0001, − 0.0001)	− 0.0001 (− 0.0002, − 0.0001)
Non-Hispanic Blacks	0.0000 (− 0.0001, 0.0001)	0.0001 (0.0000, 0.0001)	− 0.0000 (− 0.0001, 0.0001)
Mexican Americans	− 0.0001 (− 0.0001, − 0.0000)	− 0.0000 (− 0.0001, 0.0000)	− 0.0001 (− 0.0002, − 0.0000)
Other Races	0.0000 (− 0.0000, 0.0001)	0.0001 (0.0001, 0.0002)	0.0001 (0.0000, 0.0002)

Model 1: no covariates were adjusted. Model 2: age and race were adjusted. Model 3: age, race, dietary intake within 24 h (protein, fat and dietary fiber), body mass index, income poverty ratio, total protein, blood urea nitrogen, total cholesterol, serum phosphorus and serum calcium were adjusted. In the subgroup analysis stratified by race, the model is not adjusted for race, respectively

## Discussion

This cross-sectional study was conducted to clarify the relationship between fibrinogen and total BMD by performing multivariate linear regression analysis on NHANES data from 1999 to 2002. Our study primarily showed that fibrinogen levels were negatively associated with total BMD in most postmenopausal women aged 50 years and older. However, this association varies by race. To the best of our knowledge, the relationship between fibrinogen and total BMD in postmenopausal women is still poorly studied and the mechanism of the negative association is unclear.

Chronic inflammation has been identified as a predisposing factor for progressive bone loss [8, 9]. People with significantly higher levels of inflammatory cytokines than healthy individuals are more prone to osteoporosis, and accumulation of inflammatory cytokines can mediate oxidative stress injury, promote osteoclast proliferation, and increase bone resorption, leading to osteoporosis [10, 11]. Inflammation is usually accompanied by elevated levels of pro-inflammatory mediators such as interleukin-1β (IL-1β), tumor necrosis factor-α (TNF-α), C-reactive protein (CRP), and interleukin-6 (IL-6) and their markers, reflecting destructive and degenerative processes in tissues [12]. These peripheral blood biomarkers, have been used to monitor disease states and assess treatment outcomes [13]. It is becoming clear that fibrinogen appears to be one of the predisposing factors for low-grade chronic inflammation. In addition to the role of fibrin in coagulation, excessive deposition of fibrin in the tissue matrix can lead to increased local inflammation. In an experimental animal study, it was found that severe osteoporosis, which is fibrinogen-dependent, disorders of bone remodeling units with fibrinogen-mediated osteoclast activation occurred in mice defective in the fibrinogen gene [14]. However, a study by Cole et al. [7] found impaired fibrinolysis due to fibrinogen deficiency, leading to persistent fibrin deposition, resulting in premature skeletal aging and growth retardation. Increased fibrinogen promotes osteoclast activation, and fibrinogen regulates actin organization through its effects on osteoclast morphology; it not only increases actin organization and enhances bone resorption, but also promotes the formation of M-CSF and RANKL-induced bone resorption, and it alone induces bone resorption in vitro [15]. Thus, maintenance and development of the skeletal vascular system require adequate fibrinolytic activity to prevent premature skeletal aging and developmental retardation. Past studies have typically explored the effects of estrogen or inflammation on bone density alone, and there is no evidence that estrogen may contribute to osteoporosis by affecting inflammation levels [16]. Postmenopausal women will experience an increase in inflammatory cytokines due to a decrease in estrogen, which will cause excessive osteoclast production and bone resorption [17, 18]. Our study suggests that fibrinogen is negatively associated with total BMD. Fibrinogen deposition may increase the risk of bone loss by promoting chronic inflammation providing a new direction for research on the mechanisms of osteoporosis.

Interestingly, relatively high levels of fibrinogen have been found to be associated with lower BMD only in Non-Hispanic White and Mexican American postmenopausal women. This may be related to dietary habits and body fat distribution in different races, especially ectopic and visceral fat [9]. Some experimental animal studies have found that high-fat diet-induced obesity increases the deleterious effects of estrogen deficiency on bone in a mouse model of postmenopausal women, and that obesity and estrogen deficiency may affect inflammation levels leading to increased bone fragility [19]. The higher fat intake in the modern diet has led to a higher proportion of polyunsaturated fatty acids (PUFA), resulting in low-grade chronic inflammation (LGCI), which promotes the development of many chronic diseases, including obesity and osteoporosis [20]. Therefore, osteoporosis is also considered to be a chronic inflammatory disease [21]. Fibrinogen has rarely been used as a predictor of changes in bone mineral density. In previous studies, evidence for a direct correlation between fibrinogen and total BMD is very limited. Therefore, we conducted this study. In the present study we found a negative correlation between fibrinogen and total BMD. If serum fibrinogen concentration is used as a routine indicator in clinical investigations, it would be clinically relevant for most patients to assess the risk of reduced bone mineral density based on these findings.

**Fig. 1:**
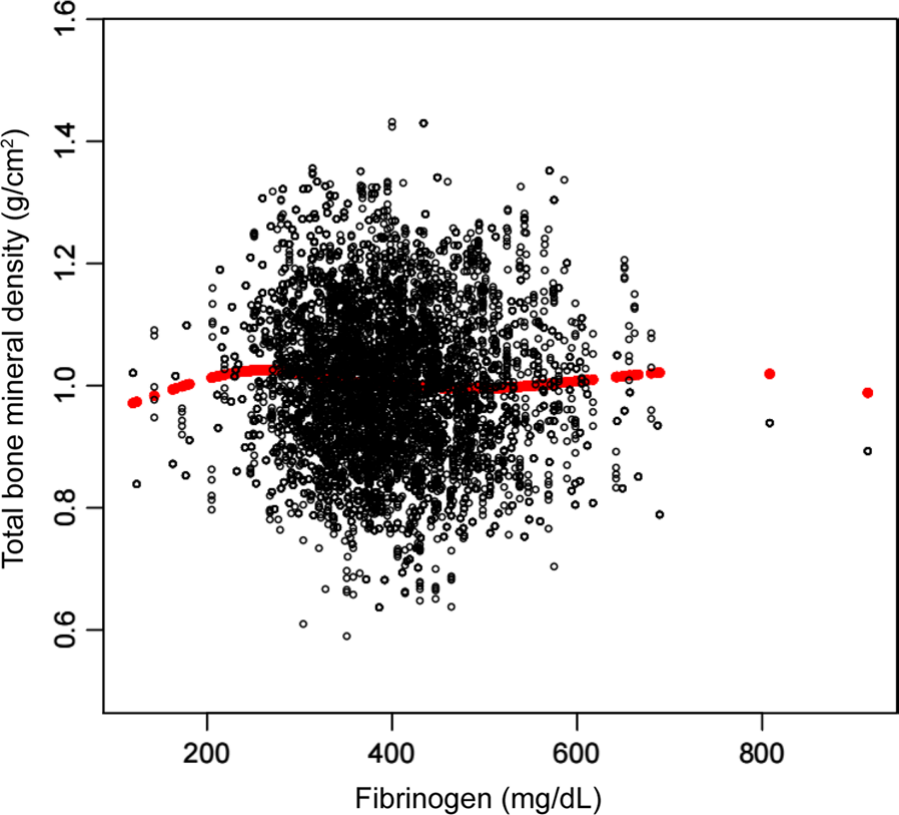
The association between fibrinogen and total BMD. Each black dot represents a sample. Age, race, dietary intake within 24 h (protein, fat and dietary fiber), body mass index, poverty to income ratio, total cholesterol, total protein, blood urea nitrogen, phosphorus, and serum calcium were adjusted. The red curve is a smooth curve fitting to the scatter diagram, representing the relationship between total BMD and fibrinogen level

**Fig. 2:**
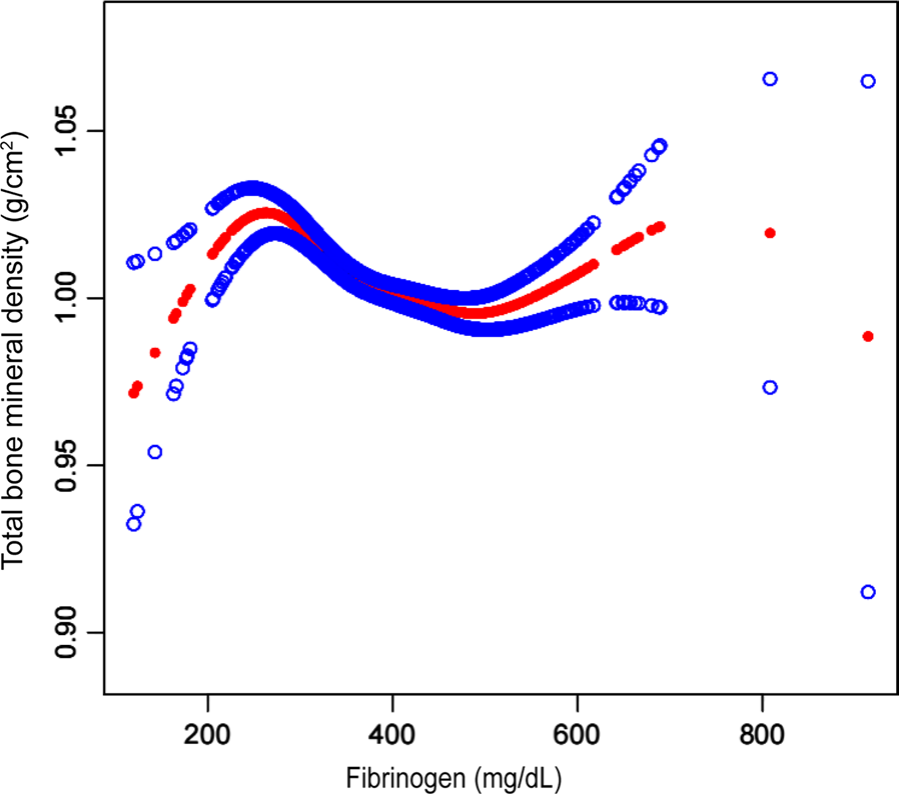
The association between fibrinogen and total BMD. The solid arcs indicate the smoothed curve fit between the variables. The blue bars represent the fitted 95% confidence intervals. Age, race, dietary intake within 24 h (protein, fat and dietary fiber), body mass index, poverty to income ratio, total cholesterol, total protein, blood urea nitrogen, phosphorus, and serum calcium were adjusted

**Fig. 3:**
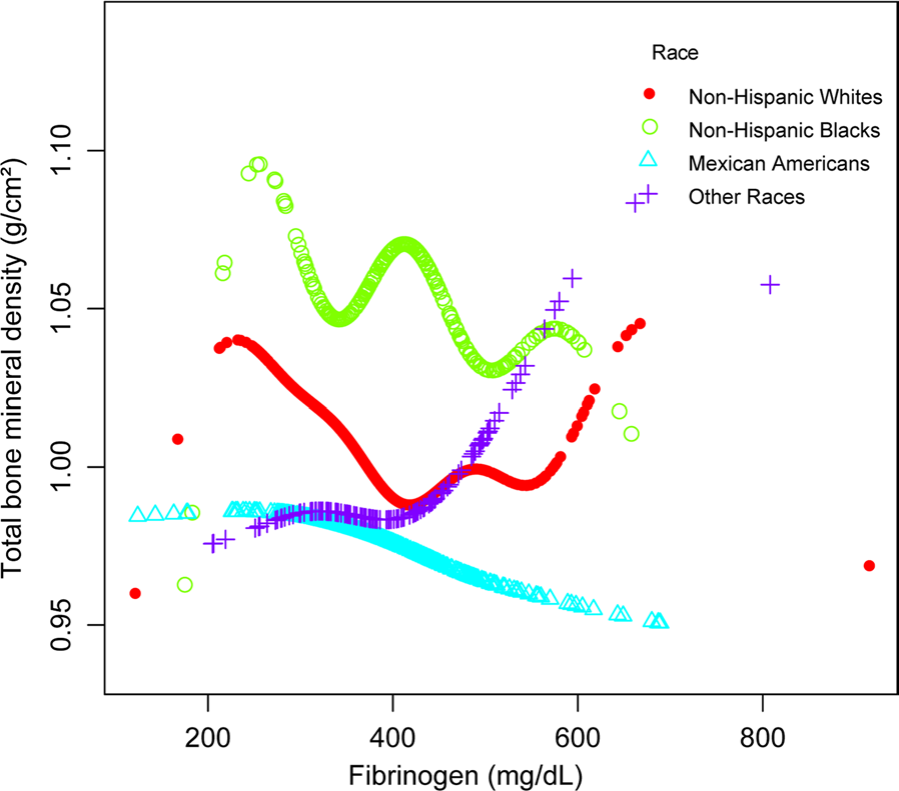
The association between fibrinogen and total BMD stratified by race. Age, dietary intake within 24 h (protein, fat and dietary fiber), body mass index, poverty to income ratio, total cholesterol, total protein, blood urea nitrogen, phosphorus, and serum calcium were adjusted

However, there are some shortcomings in our study. First, because the present study was a cross-sectional study, it was difficult to determine whether there was a causal relationship between fibrinogen and bone mineral density. Second, other confounding factors not included in the present study may also lead to bias. Therefore, we need to conduct a longitudinal study with a large sample to determine the relationship between fibrinogen and bone mineral density in postmenopausal women (Fig. 1, 2, 3).

## Conclusion

In conclusion, the relationship between fibrinogen levels and total BMD varies by race. For most postmenopausal women in the United States, relatively high fibrinogen levels may be detrimental to bone health, whereas for individuals identifying as Other Races, relatively high fibrinogen levels may be beneficial to bone health.

## Data Availability

Not applicable.
